# Physiological and growth response of rice plants (*Oryza sativa* L.) to *Trichoderma spp*. inoculants

**DOI:** 10.1186/s13568-014-0045-8

**Published:** 2014-05-29

**Authors:** Febri Doni, Anizan Isahak, Che Radziah Che Mohd Zain, Wan Mohtar Wan Yusoff

**Affiliations:** 1 School of Biosciences and Biotechnology, Faculty of Science and Technology, Universiti Kebangsaan Malaysia, 43600 Bangi, Selangor, DE, Malaysia; 2 School of Environmental and Natural Resources Sciences, Faculty of Science and Technology, Universiti Kebangsaan Malaysia, 43600 Bangi, Selangor, DE, Malaysia

**Keywords:** Trichoderma spp, Rice, Physiological response, Growth response

## Abstract

*Trichoderma spp*., a known beneficial fungus is reported to have several mechanisms to enhance plant growth. In this study, the effectiveness of seven isolates of *Trichoderma spp*. to promote growth and increase physiological performance in rice was evaluated experimentally using completely randomized design under greenhouse condition. This study indicated that all the *Trichoderma spp*. isolates tested were able to increase several rice physiological processes which include net photosynthetic rate, stomatal conductance, transpiration, internal CO_2_ concentration and water use efficiency. These *Trichoderma spp*. isolates were also able to enhance rice growth components including plant height, leaf number, tiller number, root length and root fresh weight. Among the *Trichoderma spp*. isolates, *Trichoderma sp*. SL2 inoculated rice plants exhibited greater net photosynthetic rate (8.66 μmolCO_2_ m^−2^ s^−1^), internal CO_2_ concentration (336.97 ppm), water use efficiency (1.15 μmoCO_2_/mmoH_2_O), plant height (70.47 cm), tiller number (12), root length (22.5 cm) and root fresh weight (15.21 g) compared to the plants treated with other *Trichoderma* isolates tested. We conclude that beneficial fungi can be used as a potential growth promoting agent in rice cultivation.

## Introduction

Rice growth is influenced by a combination of genotype, environment and management factors. Balancing and improving soil fertility is one of the main factors in enhancing rice growth and yield. The intensive cultivation of rice that depends on chemical fertilizers and pesticides have led to the decrease in soil fertility and deteriorating soil health. The excessive use of chemical fertilizers in the current decades has led to soil toxicity through the presence of toxic heavy metals and adversely affecting the health of rice plants (Habibah *et al*. [2011]). To overcome the decrease in soil fertility and deterioration in soil health caused by the use of chemical fertilizers, it is necessary to look for alternative ways to improve soil fertility and stimulate the growth of rice plants.

Microbes have been reported to be a key factor in maintaining soil quality and increasing rice yield and growth. The use of microbes to enhance rice growth while making the plant resistant to pathogens has been reported as an eco-friendly way to maintain the ecosystem. For decades, the application of microbes in a sustainable agroecological manner has increased rapidly due to their ability to act as plant growth promoters (Sakthivel and Gnanamanickam [1987]; Carreres *et al*., [1996]; Malik *et al*., [1997]; Anhar *et al*., [2011]; Pedraza *et al*., [2009]; Amprayn *et al*., [2012]; Anizan *et al*., [2012]).

*Trichoderma spp*. recently was suggested as a Plant Growth Promoting Fungi (PGPF) due to their ability to produce siderophores, phosphate-solubilizing enzymes, and phytohormones (Doni *et al*., [2013]). *Trichoderma* species play important roles in decomposition, mycoparasitism, and even in cellulose degradation (Jiang *et al*., [2011]; Druzhinina *et al*., [2006]; Samuels, [1996]). *Trichoderma spp*. was reported to be able to increase growth in plants such as strawberries, tomatoes, soya beans, apples, cotton and gray mangroves (Saravanakumar *et al.,*[2013]; Raman, [2012]; John *et al*., [2010]; Shanmugaiah *et al*., [2009]; Morsy et al. [2009]; Porras *et al*., [2007]). However, very little research has been conducted on the potential of *Trichoderma spp*. for improving rice growth.

Recently, we reported the successful isolation of several *Trichoderma spp*. and which have been proven to possess positive plant growth promotion potential in enhancing rice seed germination and vigour (Doni *et al*., [2014]). This research was conducted to examine the effect of *Trichoderma spp*. on rice growth and physiological response.

## Materials and methods

This experiment was conducted at the Fermentation Technology Laboratory, Genetics Molecule Laboratory and Greenhouse, School of Biosciences and Biotechnology, Faculty of Science and Technology, Universiti Kebangsaan Malaysia. A completely randomized design (with nine treatments and three replications) was used for this experiment. Rice plants were placed in soil with different *Trichoderma spp*. inoculants and soil with NPK fertilizer 100 kg ha^−1^. The NPK fertilizer used is in accordance to the rate employed in Micheal *et al.*, ([2013]) for rice variety MRQ74. Sterilized soil without any application was used for control.

### *Trichoderma spp*. inoculants preparation

Local isolates of *Trichoderma spp.* which was isolated from System of Rice Intensification Paddy Field, Sik, Kedah, Malaysia namely *Trichoderma sp.* SL1, *Trichoderma sp.* SL2 (Public Accession number: UPMC 1021), *Trichoderma sp*. SL3, *Trichoderma sp*. SL4, *Trichoderma sp*. SL5, *Trichoderma sp*. SL6 and *Trichoderma sp.* SL7 were obtained from the Fermentation Technology Laboratory, School of Biosciences and Biotechnology, Faculty of Science and Technology, Universiti Kebangsaan Malaysia. Each isolate was grown separately in potato dextrose broth and incubated for seven days in a thermo shaker at a speed of 200 rpm and 30°C. After incubation, *Trichoderma spp*. inoculants were filtered using filter paper and stored in sterile polyethylene plastic bags.

### Soil preparation and *Trichoderma spp*. inoculation

Autoclaved homogenous sandy clay loam soils were used for this experiment; the dosage of *Trichoderma spp*. inoculants was set at 5 g per 1 kg soil. The inoculated soil was immediately placed in 15 × 15 cm plastic containers.

### Rice plant preparation

Rice seeds of the variety MRQ74 were surface sterilized with 70% ethanol, followed by 5% sodium hypochlorite and washed by sterilized distilled water. The rice seeds were grown in autoclaved sandy clay loam soil under green house condition with 30 ± 4°C temperature, 320 ± 3 μmol light intensity, 80 ± 3% humidity and 11 h 11 m 17 s ± 9 s photoperiod, and placed in a seedling tray. Seven day-old rice seedlings of MRQ74 were transplanted singly in 15 × 15 cm plastic containers containing different *Trichoderma spp*. treatments, NPK treatment and control. Water was maintained at 2 cm level from the soil surface and actively aerated by physically disturbing and breaking-up the soil surface once every ten days.

### Measurement of rice growth and physiological components

Rice physiological and growth components were measured 30 days after transplanting. Measurements for the physiological characteristics were made on flag leaves. Measurements of net photosynthesis (μmol CO_2_ m^−2^ s^−1^), leaf stomatal conductance (mol H_2_O m^−2^ s^−1^), and transpiration rate (mmol H_2_O m^−2^ s^−1^) were monitored using a LICOR 6400 portable photosynthesis system (Lincoln, Nebraska, USA) and infrared gas analyser (IRGA). To assess the trade-off between CO_2_ uptake and water loss, instantaneous water-use efficiency (WUE) was calculated as the ratio between photosynthetic rate and transpiration rate (μmol CO_2_/μmol H_2_O). These measurements were taken on a clear sunny day between 09:45 am until 11:30 am under a saturated light condition (solar radiation > 1200 μmol.m^−2^.s^−1^). Plant height (cm) measured from ground level to the tip of the longest leaf, tiller number and leaf number were counted for each treatment and control. For rice root length (cm) and root fresh weight (g) measurements, the rice plants were separated carefully from the soil. Root length was measured from the base of the stem to the longest root using a ruler and root fresh weight was measured using digital scales. Rice root dry weight (g) measurement was done after rice roots were dried in the oven at a temperature of 65°C for seven days.

### Statistical analyses

All data were statistically analyzed using one-way analysis of variance (ANOVA). The significance of the effect of the treatment was determined using F-test and to determine the significance of the difference between the means of the treatments, least significant difference (LSD) was calculated at 5% probability level. Regression relationship was determined using the data analysis SPSS software version 20.

## Results

### Rice plant growth performance

The results of the experiments showed that the treatment of rice plants with *Trichoderma spp*. has a significant effect on rice plant growth performance (Table 1). Significant increase in plant height was observed for the *Trichoderma spp.* treated rice plants, registering plant heights in the range of 65.77 – 70.47 cm whilst the means for NPK treated rice plants and control were recorded at 28.9 cm and 63.1 cm respectively. Significantly greater values for leaf number and tiller number were also observed for *Trichoderma spp.* treated rice plants. The highest value for leaf number was 38 whilst for tiller number was 13 for the *Trichoderma spp.* treated plants. On the other hand, the NPK treated rice plant registered 3 leaf number, whilst the control recorded 28. No tiller was observed for the NPK treated rice, whilst the control registered a tiller number of 8. Root length was found to be significantly increased for the *Trichoderma spp.* treated rice plants with a range between 17.17–22.5 cm whilst the NPK treated rice plants recorded root length of only 9.33 cm. For the control, root length was 14.57 cm. Root fresh weight was significantly higher for the *Trichoderma spp.* treated rice plants as well compared to NPK treated and control. The *Trichoderma spp.* treated rice plants registered the highest value which was 15.21 g whilst the root fresh weight for NPK treated plants and control were 3.07 g and 8.51 g respectively. Among the *Trichoderma sp*. SL2 treated rice plants showed the greatest increase in plant height, tiller number, root length and root fresh weight, while *Trichoderma sp.* SL7 had the greatest effect in enhancing the number of leaves. However, no significant improvement in root dry weight was seen for the *Trichoderma spp.* treated rice plants.

**Table 1:** Comparison of plant height, leaf number, tiller number, root length, root dry weight and root wet weight in different treatments

**Treatment**	**Plant height (cm)**	**Leaf number**	**Tiller number**	**Root length (cm)**	**Root dry weight (g)**	**Root fresh weight (g)**
*Trichoderma sp*. SL1	69.23(3.29)	36(3.46)	12(1.53)	21.43(1.440)	2.68(1.39)	9.83(1.73)
*Trichoderma sp*. SL2	70.47(5.66)	37(4.93)	12(2.65)	22.5(1.76)	4.27(3.33)	15.21(3.20)
*Trichoderma sp*. SL3	65.77(1.75)	24(2.65)	9(1.15)	15.43(1.10)	1.94(1.13)	8.18(1.68)
*Trichoderma sp*. SL4	66.47(1.39)	30(5.20)	10(1.73)	17.17(0.65)	2.74(1.28)	11.57(0.49)
*Trichoderma sp*. SL5	66.50(4.30)	32(2.52)	13(3)	17.37(4.58)	2.74(0.91)	14.8(0.77)
*Trichoderma sp*. SL6	68.97(3.54)	37(2.65)	11(0)	21.9(2.71)	2.2(0.84)	12.96(3.51)
*Trichoderma sp*. SL7	67(1.15)	38(3.79)	12(1.73)	21.52(.30)	2.71(0.46)	15.01(3.38)
NPK	28.9(0.72)	3(0.58)	1(0)	9.33(0.91)	0.62(0.33)	3.07(0.76)
Control	63.1(0.96)	28(1)	8(1.53)	14.57(3.93)	1.94(0.37)	8.51(0.37)
LSD_0.05_	55.46	33.08	13.02	9.18	ns	9.57

ns: Not significant.

Standard deviations are given in parentheses (*n* = 27).

### Rice plant physiological characteristic

**Figure 1:**
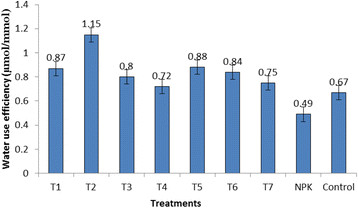
Changes in the water use efficiency by the different treatments.

The general assessment from this experiment is that the application of *Trichoderma spp*. to rice plants significantly increased the physiological properties of the rice plants (Table 2). Net photosynthetic rate was high in rice plants inoculated with *Trichoderma spp*. compared to NPK treatment and control. The range of net photosynthetic rate in rice inoculated with *Trichoderma spp*. was 6.74 – 8.79 μmol CO_2_ m^−2^ s^−1^ while NPK treatment and control recorded values of 2.09 μmol CO_2_ m^−2^ s^−1^ and 6.21 μmol CO_2_ m^−2^ s^−1^ respectively. Significantly high values of stomatal conductance were recorded for rice plants inoculated with *Trichoderma sp*. SL3 and *Trichoderma sp*. SL6 registering 1237.88 and 1084.76 mmol H_2_O m^−2^ s^−1^ respectively (Table 2). Stomatal conductance for NPK treatment and control were 340.16 and 818.30 mmol H_2_O m^−2^ s^−1^ respectively. Inoculation of rice plants with *Trichoderma spp*. also significantly influenced the internal CO_2_ concentration of the rice plants. Results showed that *Trichoderma sp*. SL2 has the lowest CO_2_ concentration (Table 2). Rice plants inoculated with *Trichoderma spp*. significantly enhanced the water use efficiency compared to NPK treatment and control (Figure 1). The highest water use efficiency was observed for *Trichoderma sp*. SL2 treated plants which registered 1.15 μmol CO_2_/mmol H_2_O.

**Table 2:** **Comparison of net photosynthetic rate, stomatal conductance, transpiration and internal CO**_**2**_**concentration in different treatments**

**Treatment**	**Net photosynthetic rate (μmolCO**_**2**_**m**^**−2**^ **s**^**−1**^**)**	**Stomatal conductance (mmolH**_**2**_**O m**^**−2**^ **s**^**−1**^**)**	**Internal CO**_**2**_**concentration (ppm)**
*Trichoderma sp*. SL1	8.79(0.010)	979.08(0.00009)	358.91(0.037)
*Trichoderma sp*. SL2	8.66(0.007)	412.40(0.00070)	336.97(0.086)
*Trichoderma sp*. SL3	8.47(0.018)	1237.88(0.0022)	363.79(0.046)
*Trichoderma sp*. SL4	6.88(0.009)	752.65(0.0024)	358.60(0.023)
*Trichoderma sp*. SL5	7.38(0.043)	499.54(0.0008)	348.35(0.172)
*Trichoderma sp*. SL6	8.60(0.012)	1084.76(0.0014)	361.75(0.039)
*Trichoderma sp*. SL7	6.74(0.024)	712.38(0.0005)	361.23(0.076)
NPK	2.09(0.007)	340.16(0.0004)	376.69(0.045)
Control	6.21(0.016)	818.30(0.0002)	365.74(0.051)
LSD_0.05_	0.10	0.005	3.70

Standard deviations are given in parentheses (n = 36).

All means were significantly different between treatments at p < 0.05.

## Discussion

### Rice plant growth performance

**Figure 2:**
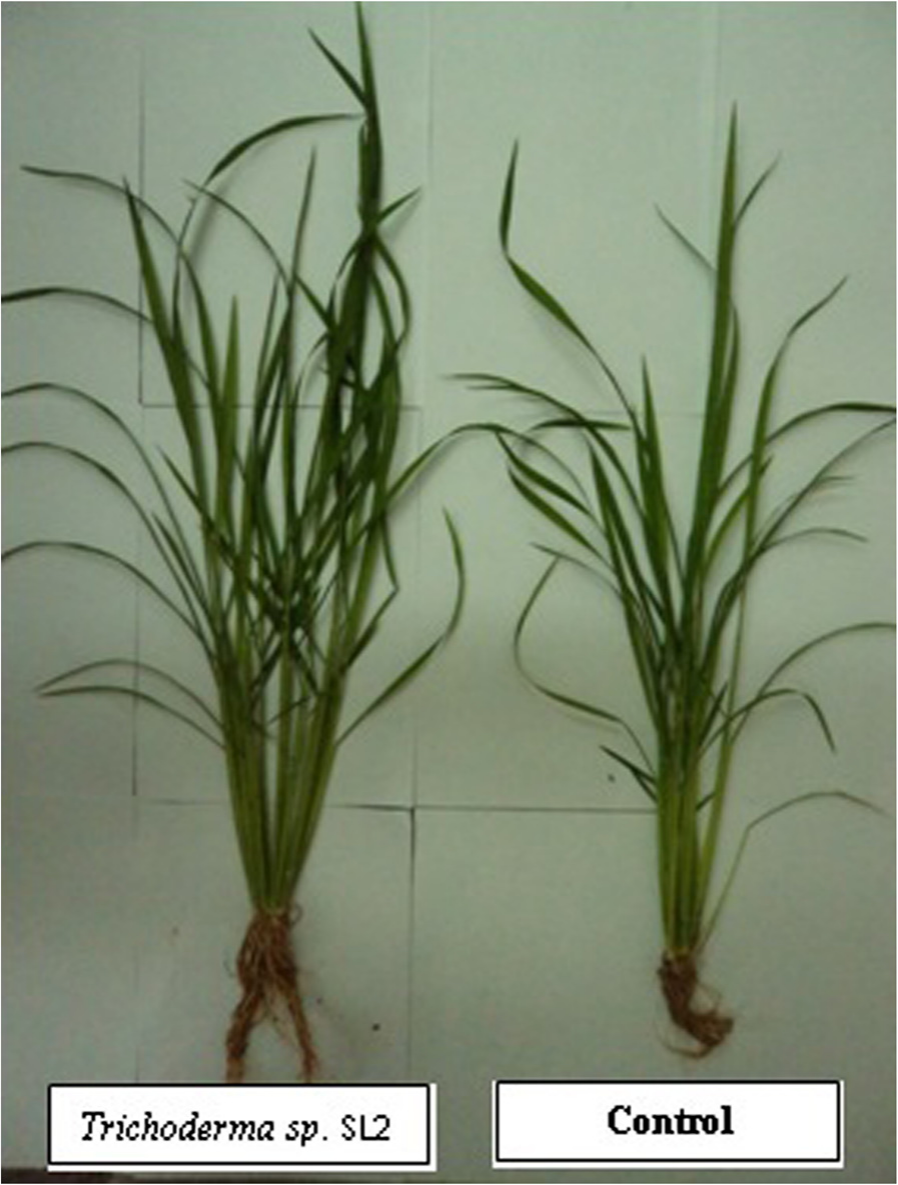
**The use of** ***Trichoderma sp.* SL2 resulted better growth compared to control.**

The benefit of *Trichoderma spp*. in improving plant growth can be realised through several mechanisms which include mycoparasitism, antibiosis, degradation of toxins, inactivation of pathogenic enzymatic pathways, resistance to pathogens, enhanced nutrient uptake, solubilization, sequestration of inorganic nutrients, and enhanced root hair development (Harman, [2006]; Lorito *et al*., [2010]). In this research we tested the ability of *Trichoderma spp*. to enhance rice growth. The results showed that rice plants inoculated with *Trichoderma spp*. significantly increased rice growth components. Plant height of *Trichoderma spp*. inoculated rice plants was higher compared to NPK treatment and control. The ability of *Trichoderma spp*. to produce phytohormones is the key factor in the increase in rice plant height as reported by Chowdappa *et al*. ([2013]). Rice plants treated with *Trichoderma spp.* also have better nutrient uptake as suggested by Saba *et al*. ([2012]). Better nutrient uptake will enhance the physiological processes within the rice plants treated with *Trichoderma spp.* leading to good growth performance (Figure 2). Leaf number and tiller number were significantly higher in *Trichoderma spp.* treated rice plants compared to NPK treatment and control. The enhancement of leaf and tiller number by *Trichoderma spp.* were made possible because of the ability of the *Trichoderma spp.* to act through several mechanisms such as environmental buffering (against pH, drought, waterlogging, cold and heat), P solubilization and siderophore production (Neumann and Laing, [2006]). Furthermore, Shukla *et al*. ([2012]) reported that *Trichoderma harzianum* significantly increased the ability of rice plants to tolerate drought stress and increase rice water-holding capacity. In this research, these mechanisms are believed to be contributing factors that led to higher leaf number and tiller number.

**Figure 3:**
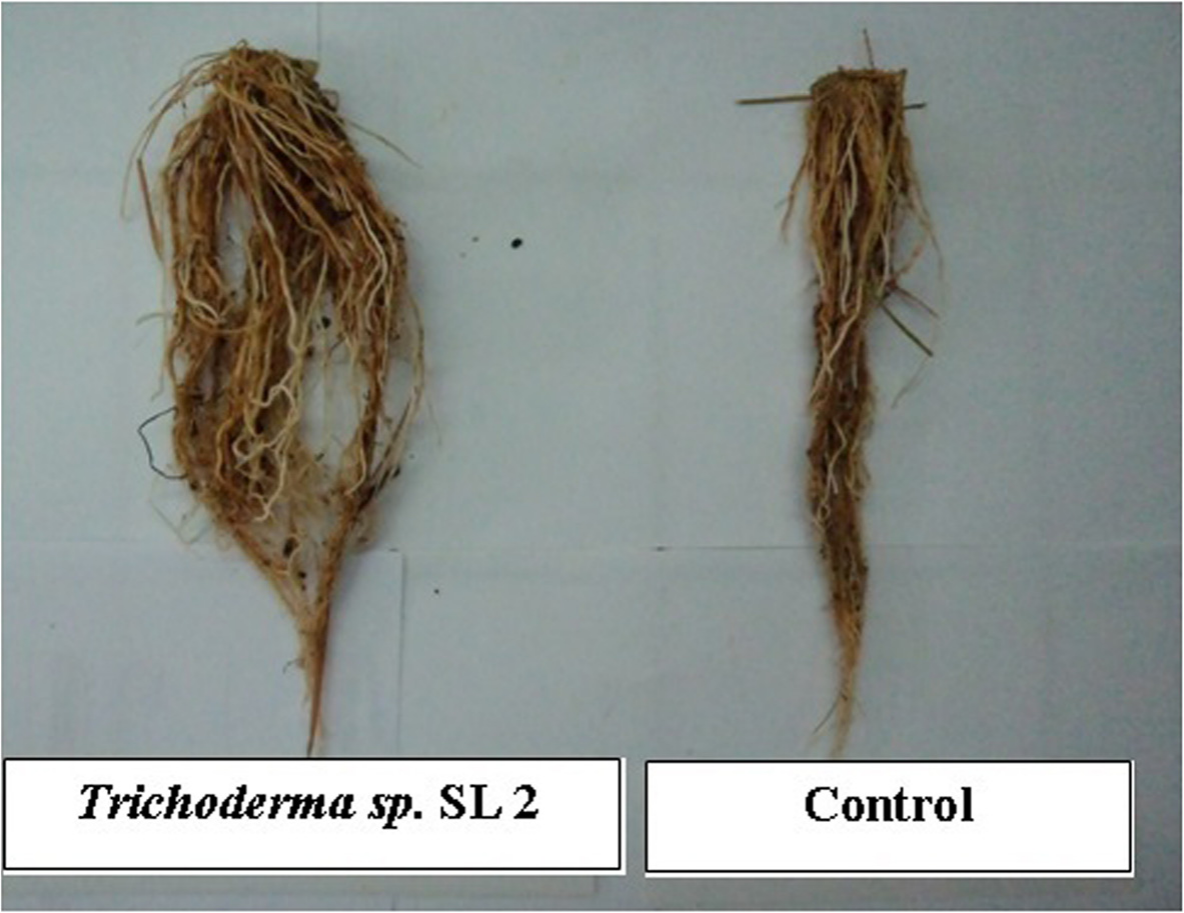
**Rice plant root inoculated by** ***Trichoderma sp*. SL 2 recorded large mass compared to control.**

*Trichoderma spp*. applied to rice plants reported in this research significantly increased rice root length compared to NPK treatment and control (Figure 3). *Trichoderma sp*. SL2 treated rice plants showed an impressive increase in root length compared to the plants treated with the other strains. Nawrocka and Malolepsza ([2013]) stated that the ability of *Trichoderma spp*. hyphae to release elicitors may contribute to signals being transmitted within the plant such as salicylic acid (SA), jasmonic acid (JA) and reactive oxygen species (ROS). Elicitors released by *Trichoderma spp.* are also involved in triggering expressions of defense protein within the plant (Thakur and Sohal, [2013]). In this way, plant immunity against pathogens is induced and in turn improves plant growth. Tchameni *et al*. ([2011]) showed that the inoculation of *Trichoderma spp.* in cacao plants may induce cacao plant resistance against *Phytophthora megakarya* and increase cacao root length. In addition, Cai *et al*. ([2013]) reported that harzianolide produced by *Trichoderma spp*. can improve the early stage of plant development through the enhancement of root length.

The fresh weight of rice root plants treated with *Trichoderma spp*. was significantly greater than the NPK treated plants and control. However, the weight of dry rice root plants was not significant among treatments. Rice plants inoculated with *Trichoderma sp*. SL2 have the highest increase in root fresh weight. The capacity of *Trichoderma spp*. to produce growth hormones such as auxins and gibberelins were reported as the main factor that contributes to the ability of *Trichoderma spp.* to support root growth and increase water absorption from soil (Arora *et al*., [1992]; Contreras-Cornejo *et al*., [2009]; Martínez-Medina *et al*., [2011]). The present findings are also in agreement with previous research by Viterbo *et al*. ([2010]) that revealed the role of *Trichoderma asperellum* in promoting canola seedling root elongation via ACC deaminase (ACCD) activity.

Our study also reports a lack of response of the rice variety MRQ74 to NPK fertilizer. Recently, research done by Khairiah *et al*. ([2013]) revealed that the heavy metal content of the rice variety MRQ 74 plants in chemical fertilizers-used rice field area was high due to excessive usage of NPK fertilizers. Previous research by Yap *et al*. ([2009]) also suggested that the use of NPK fertilizers in rice cultivation significantly increase heavy metal content in rice plants. Further, Yadav ([2010]) stated that heavy metals may cause oxidative stress inside the plants leading to cellular damage. Our research is in agreement with previous researches which revealed the lack response of this variety to NPK fertilizer.

### Rice plants physiological characteristics

Rice growth performance is subjected to environmental factors which affect the physiological processes inside rice plant cells. Improving rice physiological characteristics is considered to be desirable due to its agronomic importance towards the achievement of high rice yield (Makino, [2011]; Li *et al*., [2012]). From Table 2, net photosynthetic rates, stomatal conductance, and internal CO_2_ concentration were significantly different between treatments. High photosynthetic rates were observed for *Trichoderma sp.* SL1 and *Trichoderma sp.* SL2. It is known that large amounts of sucrose exudates provided by rice roots to *Trichoderma sp*. SL1 and *Trichoderma sp*. SL2 can facilitate good root colonization by *Trichoderma spp*. as well as promote good coordination of defense mechanism of rice plants (Vargas *et al*., [2009]). The results imply that the root physiological activity of *Trichoderma sp.* SL2 is the highest among the *Trichoderma spp.* studied. The results also revealed that *Trichoderma sp.* SL2 has the lowest internal CO_2_ concentration and the highest photosynthetic rate. This indicates that the activity of carboxylation by CO_2_ fixation for glucose production as carbohydrate metabolism in the rice plants was very active. The results agree with the findings of Thakur *et al*. ([2010]).

Research by Thakur *et al*. ([2010]) found that high photosynthetic rates coupled with low transpiration rates indicate high water use efficiency. This report supports our experiment on *Trichoderma sp.* SL2 which showed high values for these parameters. The activity of *Trichoderma spp*. that contributes to the enhancement of root growth and distribution was also considered as a key factor to the prolonged photosynthetic activity and the delayed senescence in rice plants (Mishra and Salokhe, [2011]). Further, *Trichoderma spp*. was recently reported as having the potential to degrade cellulose (Jiang *et al*., [2011]). Cellulose degradation may release a large amount of N in rice plant rhizosphere. High N concentration uptake has positive correlation with photosynthetic rate.

In Figure 2, water use efficiency of *Trichoderma sp.* SL2 was the highest among the *Trichoderma spp.* isolates registering at 1.15 μmolmmol^−1^. Stomatal conductance was significantly correlated with photosynthesis. Stomatal conductance plays an importance role in generating photosynthesis in rice plants because H_2_O and CO_2_ which are involved in photosynthetic process must pass through the stomata before they enter mesophyll cells and chloroplast stroma (Fu *et al*., [2008]). Further, Harman *et al*. ([2004]) stated that mechanisms employed by *Trichoderma spp*. in enhancing nutrient availability by solubilization and chelation of minerals can increase plant metabolism leading to the enhancement of plant physiological activity.

The present study concludes that *Trichoderma spp*. have the potential to enhance rice physiological processes and growth. In this respect, the present experiment proved that *Trichoderma sp*. SL2 was the best strain compared to six others strains.

## Competing interest

All the authors declare that they have no competing interest.

## Authors’ contributions

FD and AI carried out the green house study and conducted the statistical analysis, and CRCMZ and WMWY drafted the manuscript. All authors read and approved the final manuscript.
